# Visual evoked potentials in a diagnosis of a visual pathway dysfunction of a child with an arachnoid cyst

**DOI:** 10.1007/s10633-014-9468-4

**Published:** 2014-11-15

**Authors:** Joanna Karaśkiewicz, Wojciech Lubiński, Krzysztof Penkala

**Affiliations:** 1 Department of Ophthalmology, Pomeranian Medical University, Ul. Powstańców Wielkopolskich 72, 70- 111 Szczecin, Poland; 2 Department of Systems, Signals and Electronics Engineering, Faculty of Electrical Engineering, West Pomeranian University of Technology, Szczecin, Poland

**Keywords:** Flash visual evoked potentials, Intracranial hypertension, Arachnoid cyst

## Abstract

**Purpose:**

The aim of this case report was to demonstrate the usefulness of the flash visual evoked potentials in monitoring the effects of intracranial hypertension in a preterm-born child with a congenital arachnoid cyst.

**Methods and results:**

At baseline, abnormalities were found in the right eye: exotropia and lack of foveal fixation. Visual acuity was not achieved. Pupillary responses were normal in both eyes. There was no evidence of nystagmus. Flash visual evoked potentials were normal and equal in both eyes. When repeated one year later the signal had deteriorated in both eyes; the peak times of N2 and P2 had increased. The increased VEP latencies were the only ocular signs noted. After referral to neurosurgery, intracranial hypertension was found and a shunt was performed.

**Conclusions:**

Flash visual evoked potentials may be a valuable test in monitoring patients with arachnoid cysts.

## Introduction

Arachnoid cysts (ACs) are fluid collections in the central nervous system formed as splitting or duplication of the arachnoid layer [1]. They are mostly congenital and discovered in early childhood by ultrasound screening [2]. There is a variety of AC manifestations such as increased intracranial pressure (ICP), hydrocephalus, headaches, hemiparesis and ataxia. Ocular manifestations such as papilledema, optic nerve hypoplasia, nystagmus and/or oculomotor palsy [3] may be present. There may be no indication to treat ACs in the absence of the above ocular manifestations [4]. This early recognition of ocular manifestations is a challenge for the physicians. In this study, we describe a case of a child with AC where a significant deterioration of flash visual evoked potentials (fVEPs) responses in both eyes prompted neurosurgical consultation. After an intracranial hypertension was revealed, a successful treatment by a shunt was involved.

## Case report

**Fig. 1:**
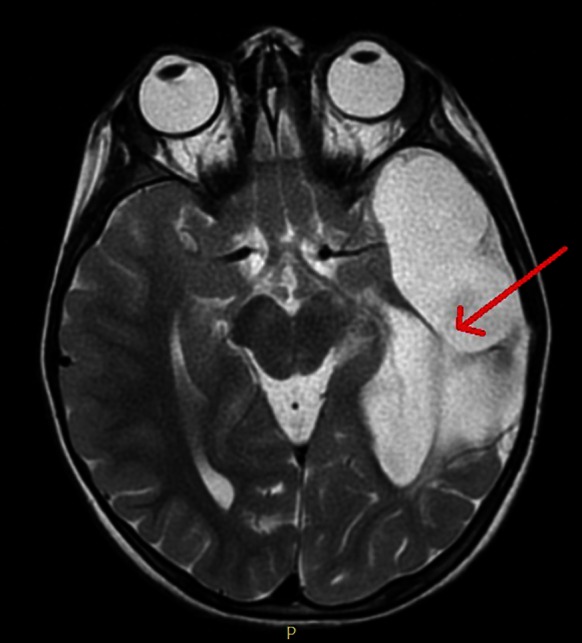
CT scan showing an arachnoid cyst (62 × 28 × 59 mm) between the temporal, parietal and frontal lobes

A three-month-old child, born in 32nd week of twin pregnancy, with a diagnosis of congenital AC in the left hemisphere (Fig. 1) was referred for ophthalmological evaluation. On a routine examination, anterior segments and stereoscopic fundus examination were normal in both eyes. Visual acuity (VA) was too difficult to perform. Pupillary responses were normal. There was no evidence of nystagmus or strabismus.

According to the neurologist’s consultation, apart from hemiparesis of the right side, the child’s condition did not raise any objections. The child was under the care of pediatric neurology and at 8 months of age magnetic resonance imaging (MRI) revealed an expansion of the AC. After an unsuccessful endoscopic fenestration of the AC, craniotomy and connection of the cyst with a ventricular system was performed by the neurosurgeon. At the age of 14 months, the child’s mother noticed an intermittent exotropia in the right eye and the child was referred to ophthalmology. On a routine ophthalmological examination, VA was not reliable. In the right eye, manifest exotropia was present and fundus examination revealed a lack of foveal fixation. In the left eye, the anterior and posterior segments were normal. To check a visual pathway function, the ophthalmologist referred the child to the electrophysiological laboratory to perform fVEPs. This test was registered according to the International Society for Clinical Electrophysiology of Vision (ISCEV 2009) [5]. Protocol of the fVEP test was implemented in the version 3.10 software of the UTAS-E 2000 system, and then modified (updated), because the original protocol did not fulfill the requirements of ISCEV fVEP standard [5]. The fVEPs tests were performed in a dimly illuminated room; monocular stimulation was used, and pupils were not dilated. Parameters of the flash stimulation (Ganzfeld, Grass Flash) were as follows: white flash, the strength, i.e., time-integrated luminance of the flash stimulus: 1.6 cd s/m^2^ (this is lower that the standard, which recommends 2.7–3.3 cd s/m^2^); background luminance: off. The frequency of the flash was 1 Hz. Central fixation light was used in the Ganzfeld dome.

## Pattern VEPs were not attempted because of the lack of cooperation with the child

**Fig. 2:**
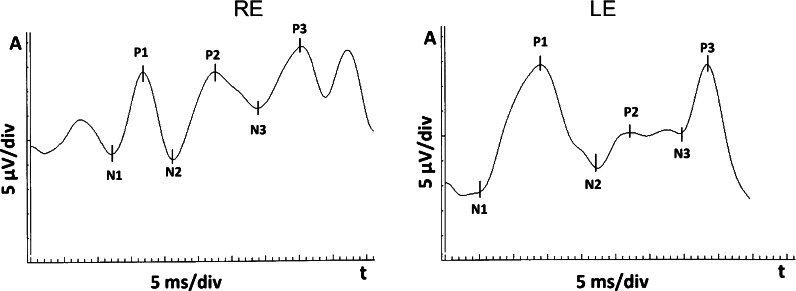
fVEPs: right eye (RE) and left eye (LE) within normal limits

**Fig. 3:**
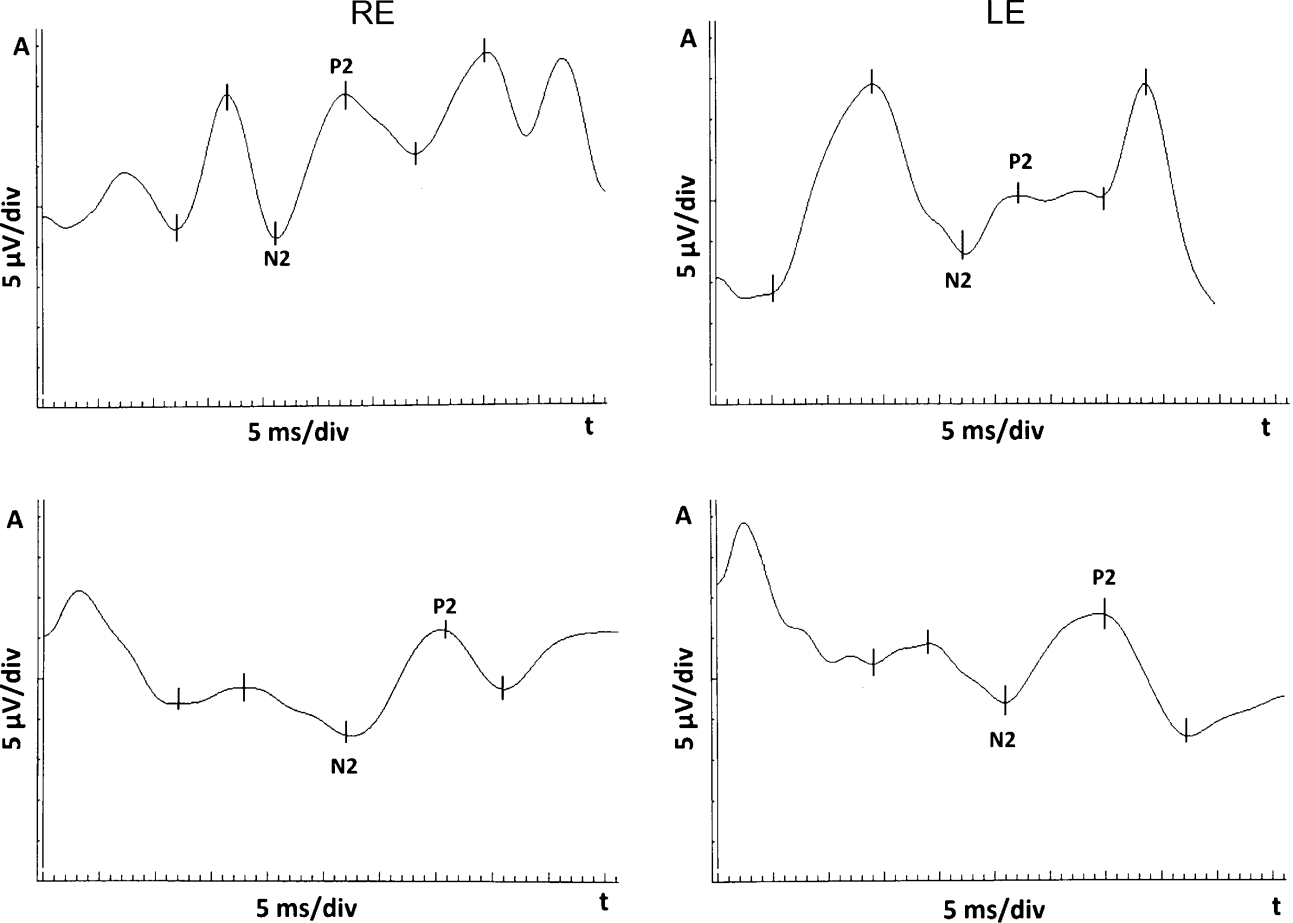
fVEPs: peak time increase of N2 and P2 waves (below) in comparison with the recording 1 year before (above)

**Fig. 4:**
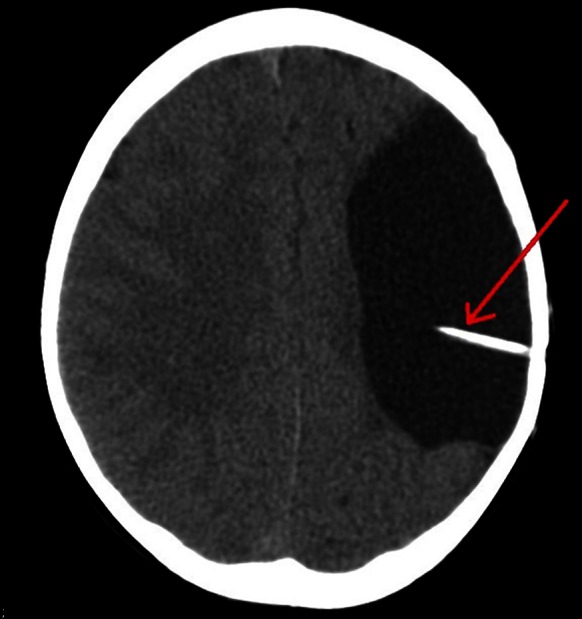
CT scan showing the shunt inside of the arachnoid cyst

Electrodes: skin electrodes (gold disk) were used; the patient’s skin was prepared by cleaning, and a suitable electrode gel (Grass) was used; the electrode impedances were below 5 kΩ; the scalp electrodes were placed on the patient’s head according to the International 10–20 system, with active electrode at Oz (or O1, O2) position, reference electrode at Fz and ground electrode at the forehead (Fpz). Parameters of the recording system were as follows: amplifiers sensitivity: 2 μV/div, filters: 0.3–100 Hz. Notch filters: off. Time base: 5 ms/div. Artifact reject threshold: 50 μV. Averaging: 80 sweeps. Results analysis: According to the standard, time parameters (peak times) of the obtained waveforms were analyzed; manual correction was applied to the automatic cursors placement. Values of all parameters were compared with own lab normal values (mean ± 2SD), which for this patient’s age are as follows: N2 peak time: 70–180 ms; P2 peak time: 95–155 ms. The fVEPs results were normal and equal in both eyes, (see Fig. 2). Follow-up fVEPs were advised after one year. After this time, at the age of 2, fVEPs were repeated. Results revealed deterioration of the signal in both eyes in comparison with the previous test; this manifested as increased peak times of N2 and P2 waves (Fig. 3). In the right eye, N2 and P2 peak times increased from 105 to 135 (29 % increase) and from 130 to 180 (33 % increase), respectively. In the left eye, N2 and P2 peak times increased from 110 to 130 (18 % increase) and from 135 to 175 (30 % increase), respectively (Table. 1). The ophthalmologist referred the child for an urgent neurosurgical consultation, because deterioration of fVEPs results might have been caused by an increased ICP. At this time, MRI revealed slight enlargement of the AC. Based on these findings, the pressure was measured directly in the cyst. It was increased and equal 20 cm H_2_O. The child underwent an uneventful shunt surgery (Medtronic Standard) (Fig. 4) and left the hospital in a good condition with a recommendation to visit the ophthalmologist and repeat the fVEPs after half a year. After 6 months, when the child was 2.5 years old, a follow-up ophthalmological examination revealed that the VA was 0.2 in the right eye and 1.0 in the left eye (Snellen table). Repeated fVEPs did not differ significantly with the previous examination. On the ophthalmological follow-up, at the age of 4, anterior segment and fundus examination of both eyes were normal and foveal fixation in the RE was present. VA in the RE increased from 0.2 to 0.6 and in the LE was stable 1.0 (Snellen table). The right eye showed an intermittent exotropia.

**Table 1:** Significant peak time increase of N2 and P2 waves (2nd fVEPs) in comparison with the recording before one year (1st fVEPs)

Eye	RE		LE	
fVEPs	Peak time			
	N2	P2	N2	P2
1st	105 ms	135 ms	110 ms	135 ms
2nd	135 ms	180 ms	130 ms	175 ms
Peak time increase (%)	29	33	18	30
SD	21.2	32.5	14.1	28.3

## Discussion

We are showing a diagnostic value of fVEPs of a child with a congenital AC. Regular eye tests are recommended in this disease, because one of the ACs’ enlargement symptoms may be increased ICP, which may be revealed by ophthalmologic examination. It is well known that fVEPs are an objective method to noninvasively evaluate ICP [6, 7], and that is why the child was referred to make this test. The suspicion that exotropia in the right eye was caused by an elevated ICP was in agreement with one of the variety of ocular manifestations that ACs may present. The deterioration of fVEPs responses, followed by the size progression of the cyst in controlled MRI, enabled the neurosurgeon to surgically measure the ICP. The higher values of ICP indicated that previously made cyst connection with a ventricular system was insufficient and a shunt was implanted. Although fVEPs give less accurate results than pattern VEPs [5, 8], the lack of cooperation with the child allowed to perform the test accurately only in this method, because central fixation was not necessary. Here, it is important to notice that the initial fVEPs results were within normal limits. The most significant information was the prolongation of the N2 and P2 waves peak times in both eyes after 1 year. These changes exceeded those in the literature intersession variability of the same person (SD = 11 %) [9]. In our case, the pre-intervention and post-intervention peak times from both eyes were still increased, what showed that although a function of visual pathway did not improve after the shunt surgery, it was, however, not worse at the same time. It suggested that ICP was increased for a long time and caused a stasis of axoplasmic transport [6] what manifested by N2 and P2 peak time continuous deterioration. These abnormal fVEPs responses indicated permanent structural changes in the visual pathway. The improvement of VA in the RE at 4 years of age may be because of improved axoplasmic transport after the reduction of ICP. In the case of this child, fVEPs were the only test that registered the deterioration of visual pathway function during an intracranial hypertension and were a cause of an urgent neurosurgical treatment. fVEPs may be a valuable test in monitoring patients with ACs.
